# Tumours of the Bladder

**Published:** 1903-10-31

**Authors:** 


### TUMOURS OF THE BLADDER.

Dr. Henry G. Spooner,[1] in reviewing this subject, points out that vesical new growths are most frequently met with after middle life, and are twice as common in men as in women. The etiology is as yet unexplained, and although by some writers trauma has been given an important place with regard to the causation, Dr. Spooner's experience points to a contrary conclusion. He states that, while almost every patient can recall some injury, a sure history of trauma is not obtained in above 5 or 6 per cent. of all cases.

Excluding metastatic tumours and growths which have come to affect the bladder by extension from neighbouring organs, vesical new growths may be classified accordingly as they arise from (1) the mucosa and submucosa; (2) the muscular layers; (3) the epithelium and glands. Those which come under the first heading are mostly benign; they usually have a pedicle, and are commonly described as papillomata or villous growths. Dr. Spooner prefers to designate them under the name of fibroma papillare. These form the largest proportion of all bladder tumours. The pedicle consists of a submucous vascular connective-tissue framework, which spreads out peripherally into the epithelium-covered tufts or villi. The base of the pedicle may be small or large in circumference. These tumours may be single or multiple. They most frequently occur on the trigone or about the neck of the bladder. A notable feature of these papillomata is that other tumours may arise from them which show no villous structure. These secondary developments are classified histologically into fibromata, myxomata, and sarcomata; the last named being very rare.

There is only one form of tumour arising from the muscular layer, and that is the myoma. This is formed in the main of unstriped muscle and fibrous tissue. It is as a rule tough and pedunculated. It is generally situated at the neck of the bladder, and is hard to differentiate from a tumour of the prostate. It grows slowly, and shows little tendency to ulcerate. Of the tumours that are formed from the epithelium of the bladder carcinoma is the most frequent. It is often very difficult to differentiate this condition clinically from malignant disease of the prostate.

As in other parts of the body cancer in the bladder gives rise to no symptoms in the early stages of its existence. The first sign of its presence is usually hæmaturia—a few drops of blood appear in the urine unaccompanied by any pain. As the disease advances the bleeding becomes more frequent, more copious, and lasts longer. There are certain features connected with hæmaturia due to vesical new growths which are of considerable assistance towards making a correct diagnosis. When the cause of hæmaturia is a lesion of the bladder the urine which is passed at the end of micturition contains more blood than that voided at the beginning, the reverse of what occurs when the hæmorrhage comes from the urethra. Blood may also come from the bladder in cases of tuberculosis and stone, but in these conditions as a rule the hæmaturia is more marked after active exercise and is more often accompanied by pain. Hæmorrhages caused by simple or tuberculous ulcerations and stones are not often so copious as those which are due to a tumour. Another important point is that, apart from the blood, the urine is generally normal; this is not so likely to be the case if a stone or tuberculous ulceration be present. Fragments of a growth may be found in the urine. Obstructive retention may be caused by fragments of the tumour, by clots of blood, or by the undetached tumour itself, and this last is the more to be expected since tumours of the bladder are situated mostly in the region of the trigone. In all cases a cystoscopic examination is most important, for by this means every tumour can be recognised. Then again the cystoscope alone will reveal the extent of the disease, and whether it is operable or not. It should be a rule never to operate on a patient for tumour of the bladder without first making a cystoscopic examination.

The prognosis is unfavourable in every case of new growth of the bladder. That this is so in the case of carcinoma and sarcoma goes without saying, but it is a fact that benign growths show a marked tendency to become malignant with the lapse of time. Dr. Spooner himself has reported a case where a benign growth existed for 25 years and then became malignant. Even apart from this tendency to become malignant, the simple growths menace life in the long run through hæmorrhage, cystitis and secondary kidney trouble. The treatment in all cases where it is possible is removal by operation, in most cases by the supra-pubic route, but Dr. Spooner thinks the perineal method may sometimes be usefully employed, while small pedunculated growths may be detached and removed through the urethra by the wire loop and the operating cystoscope of Nitze. He recommends the administration of urotropin in 7½ grain doses three times a day for some time before operating on the bladder.

[1] Post-Graduate, October, 1903.

